# A roadmap for ‘core concepts’ in evolutionary medicine

**DOI:** 10.1093/emph/eox026

**Published:** 2018-01-12

**Authors:** Charles L Nunn

**Affiliations:** Duke University

Evolutionary perspectives are vital to tackling a wide range of health challenges in today’s world, including emerging infectious diseases, the evolution of antimicrobial resistance, increasing prevalence of autoimmune diseases, the obesity epidemic, threats to food safety, neurodegenerative disease, cancer and more. To address this scope of diseases, evolutionary medicine is highly interdisciplinary, drawing on perspectives and approaches in ecology, evolution, biological anthropology, and global health (which itself is very interdisciplinary). Some evolutionary medicine research programs focus on understanding why humans are vulnerable to disease, whereas others aim to apply evolutionary approaches to more effectively prevent or treat disease.

Because of these broad health foci and approaches, it is challenging to distill core principles for evolutionary medicine. What are the major concepts that one must understand to enter this field of research, practice, and general understanding? For those of us who teach evolutionary medicine to undergraduates, this is particularly challenging and important to consider. It is obvious from discussions with many colleagues that our courses are as heterogeneous as the topics and approaches in evolutionary medicine itself, with professors gravitating toward their areas of familiarity and leaving gaps in our curricula. This is especially important for undergraduate courses, as this may be the only exposure that our future doctors, nurses, veterinarians, dentists and public health officials have to evolutionary medicine at a broad level.

Some crucial guidance is now available in a paper just published in *Evolution, Medicine and Public Health* by Daniel Grunspan *et al.* [1]. In this paper, the authors tackle the challenge of defining core principles in evolutionary medicine by applying the Delphi method, which is a systematic way to build consensus among experts that is especially useful when a diversity of viewpoints and approaches exists. Through an iterative process of surveys, this method leads to consensus on a set of questions. Equally important, the Delphi method can reveal areas of inconsistency in responses and provide explanations for a lack of agreement.

Grunspan *et al.* [1] pursued the Delphi method via a panel of 56 experts, using four iterations of a survey that focused on the core principles in evolutionary medicine. They started by offering three different definitions of ‘core principle’, and by asking the experts to ‘list the big ideas that you feel are important in the field of evolutionary medicine’. Through four rounds of this general approach (refining the list and specific questions for experts as the research unfolded), the team identified 14 core principles that reached at least 80% agreement, which has been used previously as a mark of ‘consensus’ in the Delphi method. You can find those core principles in the published paper [1], along with levels of consensus, and a list of other concepts that were proposed but failed to reach consensus.

Grunspan *et al.*’s [1] paper is a compelling attempt to systematically identify the core principles of evolutionary medicine, and represents an impressive amount of coordination, integration of many responses and deep thought about the breadth of evolutionary medicine research (and how to define core principles in such a broad field). The outcome will be useful for teaching evolutionary medicine courses to undergraduates, and more generally, Grunspan *et al.*’s [1] contribution helps to define the perspectives and approaches that form the foundations of evolutionary medicine.

To advance these findings, *Evolution, Medicine and Public Health* will be publishing Foundational Clinical Briefs about each of the core principles that emerged from Grunspan *et al.*’s [1] paper. These are a short-format reviews that are similar to our better known Clinical Briefs, and include examples in public health and medicine. Thus far, we have only published one Foundational Clinical Brief—Peter Ellison’s contribution on Evolutionary Tradeoffs [2], which also emerged as one of the 14 core principles from the Delphi study. If you are interested in contributing a Foundational Clinical Brief for one of the core principles, please contact me so that we can avoid overlapping effort.

**Conflict of interest:** None declared.
